# Antimicrobial Resistance Genes (ARGs), the Gut Microbiome, and Infant Nutrition

**DOI:** 10.3390/nu15143177

**Published:** 2023-07-18

**Authors:** Rufus J. Theophilus, Diana Hazard Taft

**Affiliations:** Food Science and Human Nutrition Department, University of Florida, Gainesville, FL 32611, USA

**Keywords:** antimicrobial resistance, gut microbiome, infants, toddlers, nutrition, diet, antibiotics

## Abstract

The spread of antimicrobial resistance genes (ARGs) is a major public health crisis, with the ongoing spread of ARGs leading to reduced efficacy of antibiotic treatments. The gut microbiome is a key reservoir for ARGs, and because diet shapes the gut microbiome, diet also has the potential to shape the resistome. This diet–gut microbiome–resistome relationship may also be important in infants and young children. This narrative review examines what is known about the interaction between the infant gut microbiome, the infant resistome, and infant nutrition, including exploring the potential of diet to mitigate infant ARG carriage. While more research is needed, diet has the potential to reduce infant and toddler carriage of ARGs, an important goal as part of maintaining the efficacy of available antibiotics and preserving infant and toddler health.

## 1. Introduction

Antimicrobial resistance (AMR) constitutes a major global health crisis. In 2019, about 5 million deaths worldwide were associated with AMR [1], with approximately 35,000 deaths in the United States [2]. The number of resistant infections was even higher, with more than 2.8 million resistant infections occurring in the US annually [3]. The cost of these infections is high, surpassing USD 55 billion in the US, including USD 20 billion in direct health care costs and USD 35 billion in loss of productivity [2]. Approximately USD 4.6 billion of the direct health care costs related to AMR comes from treating just the six most common multidrug-resistant infections [4]. While people of all ages can develop multidrug-resistant infections, infants are particularly vulnerable because use of some antibiotics is contradicted in young children and because overuse and misuse of antibiotics are common in children [5]. AMR infections in infants can have fatal consequences, with an estimated 214,000 neonates dying from AMR-linked sepsis every year [6]. By country, India loses over 56,000 newborns to AMR infections annually, Pakistan loses over 25,000 newborns to AMR infections each year, and Nigeria loses approximately 19,400 newborns to AMR infections annually [6]. Major risk factors for AMR infections in humans of all ages include chronic diseases, surgery, and the use of antibiotics [7]. Many of these risk factors occur less frequently in infants, with the exception of antibiotic use [8,9,10,11]. Infants are commonly exposed to antibiotics both indirectly (e.g., maternal antibiotic exposure during pregnancy [12,13] or at delivery [14]) and directly to treat infections. Antibiotics are routinely and frequently given during infancy [15,16], and their abuse, misuse, or improper prescription lead to AMR infection proliferation [17]. AMR infections caused by pathogens previously susceptible to an antibiotic occur because bacteria are capable of acquiring antimicrobial resistance genes (ARGs), and these ARGs act by a wide variety of mechanisms [18]. There are a wide variety of ARGs currently known to science, and nearly all antibiotics have known ARGs [19].

There are multiple means by which a pathogen can become resistant to an antibiotic. Least concerning are pathogens that are innately resistant to some antibiotics, for example, *Pseudomonas aeruginosa* is intrinsically resistant to a number of different antibiotics [18]. In some cases of greater concern, pathogens become resistant after exposure to antibiotics by acquiring point mutations in chromosomal genes, as has occurred in some *Salmonella enterica* strains [20]. Most concerning to public health is the ability of bacteria to acquire ARGs through horizontal gene transfer (HGT), as HGT enables the rapid evolution and spread of multidrug-resistant bacteria [21,22]. The ability of ARGs to spread by HGT complicates approaches to controlling the spread of AMR infections. HGT of plasmids and transposons permits ARGs to be shared between diverse species, especially when communities of microbes are exposed to antibiotics [23]. Bacteria with ARGs on transferable genetic elements are found in many microbial communities, including human-associated communities, and myriads of animal-associated communities [24].

ARGs are identified using a variety of techniques. Traditional methods are frequently based on bacterial culture and rely on phenotype testing to find the minimal inhibitor concentration (MIC) of antibiotics [25]. Initial identification of ARGs was completed through painstaking identification of enzymes and Sanger sequencing [26,27]. This work led to PCR- and microarray-based tests for specific ARGs [28,29]. The decreasing cost of whole-genome sequencing and whole-metagenome sequencing in combination with databases of antimicrobial resistance genes made it feasible to identify ARGs from sequencing data [30,31]. These sequencing technologies enabled the unbiased study of ARGs in microbial communities without specifically testing for selected, individual ARGs [32]. The growing number of methods available for identification of ARGs are enabling discoveries of novel ARGs and a better understanding of the ARGs in complete environmental communities [33].

The ubiquity of transferable ARGs and the improvements in our ability to track and find ARGs have given rise to the One Health concept as applied to ARGs [34,35]. According to the WHO, the One Health perspective takes an integrated, unifying approach to sustainably balance and optimize the health of humans, animals, plants, and the environment/ecosystems [36]. The goal of One Health is to help to coordinate research efforts in various fields to address global health-related challenges (such as AMR) that affect humans, animals, and their environments [37]. The complexity of AMR warrants the all-discipline and all-encompassing framework of One Health [38,39]. One Health has played a pivotal role in combatting the rise of AMR through its global advocacy and interventions [34], including interventions aimed at protecting the health of infants and young children [40]. As an example of successful One Health program to reduce child mortality, the WHO has updated its yaws eradication strategy to balance the risk of increasing azithromycin resistance with the need to protect human health from bacterial diseases [41].

The One Health perspective that multiple ecosystems must be considered to fully understand AMR does not mean that all ARGs are equal threats to public health. Zhang et al. have developed a framework to describe the threat posed to ARGs by human health [42]. In this framework, the highest-risk ARGs are those that are on mobile genetic elements, are enriched in human-associated environments, and are known to occur in high-risk pathogens [42]. Because of the importance of human-associated environments to the risk levels of ARGs, consideration of the ARGs in the gut microbiome is particularly important. The gut microbiome is defined as the assemblage of commensals, symbionts, and pathogens that inhabit the human gut [43]. The gut microbiome contains the gut resistome, defined as the collection of all ARGs contained within the gut microbiome [44]. This means that the gut serves as a repository of ARGs, with healthy individuals carrying a number of AMR organisms [45]. As the human gut microbiome is by definition a human-associated environment, this suggests that the gut resistome may be of particular importance to understanding the threat posed by ARGs to human health. This makes it critical to understand the factors that shape the human gut resistome.

## 2. Diet, Age, and Antimicrobial Resistance Gene Carriage

Diet is a major driver of the gut microbiome composition, with different diets eliciting changes in gut microbiome composition [46]. Diet also changes the metabolites produced by the gut microbiome, with marked differences in the metabolome of vegans and omnivores [47]. Dietary fiber is particularly important in influencing the metabolites produced by the gut microbiome [48]. Because the gut microbiome serves as a reservoir for ARGs and because diet helps to shape the gut microbiome, dietary changes have the potential to alter the resistome. One recent study that evaluated the association of diet and antimicrobial resistance in healthy US adults demonstrated that adults who consume diets higher in fiber carry lower levels of ARGs [49]. It remains unknown if deliberate dietary interventions in adults can alter ARG carriage; but the work by Oliver et al. does suggest that increased fiber intake might result in lower ARG levels [49] and that future work on the relationship between diet and the resistome is warranted.

Use of antibiotics in agriculture drives the proliferation of ARGs [50]. Antimicrobial agents are primarily used in animal agriculture but are used at lower levels when farming plants [51]. Because of the large quantity of antimicrobial agents used during livestock production, reduction in antibiotic use in livestock production is currently a priority to protect both human and livestock health [52]. Consumption of animal products is associated with increased risk of some AMR infections, for example, consumption of poultry is associated with increased risk of multidrug-resistant urinary tract infections in women [53]. Despite this connection between meat consumption and AMR infections, one study that compared omnivorous, vegetarian, and vegan diets did not find a clear connection between diet type and ARG carriage [54], suggesting that a more nuanced understanding of diet than just crude measures of animal product consumption and total plant fiber consumption are needed to understand the associations between diet and the resistome.

Age is important to the gut resistome, as infants carry higher levels of ARGs than their own mothers [55]. As in adults, the composition of the infant gut microbiome is also important to the infant gut resistome, such that infants with higher levels of *Bifidobacterium* carry lower levels of ARGs [56]. Therefore, this review seeks to summarize what is known about the infant gut resistome, including the potential for diet to alter the resistome in infants. We will briefly highlight the connection between diet and the gut microbiome in young children, continue with a discussion of how infants acquire ARGs (including the role of breastmilk feeding), then consider the same factors during the weaning period, and finally discuss the potential for infants to act as a source of ARGs that then transmit to others.

## 3. Diet and the Gut Microbiome of Infants (0 to 6 Months)

The WHO and allied agencies recognize how crucial an appropriate diet is to maintain the growth and development of infants; therefore, to support infant health, the WHO recommends exclusive breastfeeding until infants are six months of age [57]. After six months of age, complementary foods should be introduced but the child’s diet should include continued breastfeeding until the child is two years of age or older [57]. Exclusive breastfeeding for the first six months of life is possible because breastmilk is complete nutrition for the infant during this period [58]. Breastmilk also provides infants with antibodies that are protective against infectious diseases and pathogens [59]. Furthermore, other bioactive compounds present in breast milk, including enzymes, immune cells, and antimicrobial peptides provide additional benefits to the infant by helping to reduce risk of infection [60,61].

Diet is a crucial factor affecting the composition of the gut microbiome, especially in infants [62,63]. From the earliest days of microbiome research, scientists described marked differences between the microbes found in the feces of breastfed infants and formula-fed infants [64], and these differences remain in modern infants [65]. One key difference between breast milk and formula is that breastmilk contains human milk oligosaccharides (HMOs) [66], compounds that are abundant in human milk and contribute to the formation and regulation of the infant gut microbiome [66,67]. HMOs are indigestible by infants but instead act as prebiotics supporting the growth of beneficial bacteria, including members of genera *Bifidobacterium* and *Bacteroides* [67,68,69,70]. In addition, HMOs function as soluble “decoy receptors”, potentially blocking pathogens from infecting infants [70,71,72]. By promoting the growth of beneficial bacteria and preventing infection with pathogens, HMOs contribute to the development of a healthy and stable gut microbiome [67].

HMOs are particularly important to the infant gut microbiome as they are now known to be what was once referred to as the “bifidogenic factor” of breastmilk [73]. Historically, exclusively breastfed infants had gut microbiomes dominated by *Bifidobacterium* to the point of being near monocultures, while historically formula-fed (previously referred to as “bottle-fed”) infants had a more diverse microbiome [64]. Today, there is considerable variation in the levels of *Bifidobacterium* that colonize breastfed infants based on geography, a trend potentially related to differences in historical breastfeeding patterns [74]. As ARGs are not evenly distributed across microbial taxa [75], differences in gut microbiome composition driven by differences in diet have the potential to profoundly influence ARG carriage.

## 4. Infant Gut Microbiome, ARG Acquisition, and Breastmilk Feeding

Microbial colonization of the infant gut begins at birth [76]. This initial exposure to maternal microbes forms the basis of the infant gut microbiome [77]. During vaginal birth, infants are exposed to various microorganisms from the mother’s vaginal tract and to various fecal microbiota as well as additional microbes from the immediate environment of the delivery room [77]. Infants born by Cesarean section (C-section) are not exposed to maternal vaginal or fecal microbes to the same extent but instead acquire bacteria from maternal skin and the hospital environment leading to a distinct gut microbiome composition by delivery mode [77]. Regardless of delivery mode, infants are exposed to a mixture of antibiotic-sensitive and -resistant bacteria [13,78]. The birth process therefore contributes to the vertical transmission of resistant organisms [79].

Intrapartum antibiotic use may also contribute to the vertical transmission of ARGs at birth. C-section-delivered infants are frequently exposed to intrapartum antibiotics [13,55]. This practice can reduce the risk of infection but also contributes to the selection and proliferation of resistant bacteria in infants and mothers [80]. Intrapartum antibiotic exposure use is not limited to C-section deliveries, as intrapartum antibiotic prophylaxis (IAP) is given to reduce the risk of early-onset sepsis in infants born to Group B Streptococcus-positive mothers [81]. Maternal carriage of Group B Streptococcus is common, leading to antibiotic exposure in 25% of vaginally born US infants [82]. Although this approach is effective in reducing early-onset sepsis, the downside is that it increases ARG levels in infant gut microbiome [13].

After delivery, antibiotic use by infants is common during the first year of life, with 79% of infants in a large Tennessee cohort having at least one antibiotic prescription filled during the first year of life [83]. In Europe, where antibiotic prescription is less common in the first year of life, one study reported that only 39% of European infants received at least one prescription by age 1 year [84]. Studies have found that infants who were given antibiotics early in life had greater abundance of ARGs in their gut microbiome compared to infants who were not exposed to antibiotics [78,85,86,87]. This indicates that in early life, use of antibiotics can disrupt infant gut microbiome, resulting in reduced bacterial diversity and greater carriage of resistant bacteria [78,85,86,87].

Even in the absence of antibiotic exposure, infant acquisition of ARGs does not end with delivery, with the number of different ARGs detected increasing with infant age [88]. Daycares are likely a source of antimicrobial resistance bacteria transmission, as resistant pathogens are known to spread in daycares [89]. While not specifically studied in infants, dogs are known to transmit antimicrobial resistance bacteria to their owners [90], and so household pets are also a potential source of ARGs in infants, especially as households are at least occasionally the source of infections for infants [91]. ARGs may also transmit between other household members. For example, in one study, infants with a twin had a resistome more similar to their twin than to their mothers, and infant gut resistomes were no more similar to their mothers’ gut resistome than to the gut resistome of other unrelated infants [79]. This finding of minimal similarity between the infant and maternal resistome is not consistent in the literature. A different study reported that ARGs are similar between mothers and infants [92] and that there was an overlap in the ARGs present in breastmilk and the ARGs present in the infant gut microbiome [92].

There are other studies where ARGs in the infant gut microbiome appear to be associated with those in breastmilk. For example, one study was able to isolate antimicrobial-resistant *Enterococcus faecium* from breastmilk [93], while another study reported the isolation of resistant *E. faecium* and resistant *Lactobacillus* species from milk [94]. The overlap in ARGs found in breastmilk and the infant gut microbiome is not the full story on the association of breastmilk with the infant resistome because breastmilk feeding was associated with reduced gut carriage of ARGs compared to formula feeding [95]. This relationship may be partially because of the fact that breastmilk feeding supports the growth of infant commensals, such as *Bifidobacterium*, that are associated with reduced carriage of ARGs [56,95,96]. For infants older than six months of age, breastmilk is no longer adequate for all the infant’s nutritional needs. This necessitates the introduction of solid foods, although breastfeeding remains part of the recommended infant diet until the infant is age two years or older [58], and these dietary changes have the potential to result in a changing relationship between infant diet and the gut resistome.

## 5. Infant Diet during the Complimentary Feeding Period and the Gut Microbiome (Over 6 Months)

After six months of age, breastmilk is no longer nutritionally complete for infants, as growing babies need more iron and other micronutrients than are provided by breastmilk [97]. Complementary foods should be nutrient dense and should include essential nutrients such as iron, zinc, calcium, vitamins, and a variety of healthy fats to foster healthy growth and development [98]. One important measure of a healthy diet during the complementary feeding period is dietary diversity, with infants showing improved growth with a more diverse diet [99]. Complementary feeding practices vary globally, with US infants less likely to achieve an adequate minimum diet diversity by age 12 months compared to Mexican or Chinese infants [100].

Weaning affects infants physiologically and changes the morphology of the intestine [101,102]. These changes correspond with changes in the gut microbiome that may have long-term impacts on infant health [103]. One of the most important factors that affects the gut microbiome during weaning is the timing of introduction of solid foods. Studies have shown that infants who are introduced to solid foods earlier (e.g., at 4 or 5 months of age) have a different gut microbiome composition than infants who are introduced to solid foods at later time points [104,105]. However, the observed changes have been attributed to duration of exclusive breastfeeding and not the age at which infants are introduced to solids [104,105]. Early introduction of solid foods is associated with a higher abundance of Bacteroides and a lower abundance of bifidobacteria, both of which are important groups of microorganisms that play a role in immunity and metabolism [106,107,108]. The introduction of the first complementary food to infants results in rapid changes to the metabolites produced by the gut microbiome and in changes to the beta-diversity of the infant gut microbiome [109].

The type of solid foods consumed influences the gut microbiome. Data from adults demonstrate that a diet rich in fiber, fruits, and vegetables promotes a diverse and balanced gut microbiome, while processed foods and sugar have been linked to a less diverse and imbalanced gut microbiome [110]. This is because fiber-rich foods and fruits and vegetables act as prebiotics, promoting the growth of beneficial microorganisms in the gut, while processed foods and sugar are pro-inflammatory, promoting harmful microorganisms [111]. In an in vitro system that uses gut microbes collected from infant feces, the sugar composition of fibers in artificially digested foods correlated with changes in the metabolites produced and in the diversity of gut microbes [112], further supporting the connection between dietary fiber consumption, infant gut microbiome composition, and metabolite production. In infants, the introduction of complementary feeding induces significant changes in the gut microbiome, including the replacement of Bifidobacteriacea, Enterobacteriaceae, and Lactobacillaceae with taxa more commonly found in adults, such as *Clostridium* and *Bacteriodes* [113].

## 6. Complementary Feeding Period and ARG Carriage in Infants

Preventing AMR infections is particularly critical during the complementary feeding period, as infants are more vulnerable to infection as they transition away from a breastmilk-only diet [114,115]. This period of life also exhibits marked shifts in the infant resistome, with the diversity of ARGs increasing [104] but their abundance decreasing in the gut microbiome [56]. This decrease in ARG abundance is correlated with the increasing diversity of carbohydrate utilization genes [116], suggesting a potential role for the complementary diet in shaping the resistome in early life. *Escherichia* contribute the highest abundance of ARGs [116]. The prevalence of resistant *Escherichia coli* in infants increases throughout the first year of life [117], a fact that underscores that infants continue to acquire ARGs during the complementary feeding period. This means that while the decrease in abundance of ARGs is a beneficial shift in the resistome that occurs during the complementary feeding period, this period is not unambiguously beneficial as this period also represents a shift in the resistome from bacteria with intrinsic ARGs to more transferable ARGs [116]. Despite the changes to the resistome that occur during this period, little research exists that examines the connection between complementary feeding diet and ARG carriage. Given the connections between diet and ARGs in adults [49] and the correlation between increased carbohydrate utilization genes and decreased ARG carriage [116], this is an area where more research is urgently needed to understand if the selection of some optimized set of foods may also reduce infant acquisition of new ARGs during this time period.

There are additional mechanisms by which the solid food introduced to the infant diet can contribute to ARG proliferation. Agricultural animals used for food are often farmed using antibiotics extensively for the prevention of diseases and support of growth, which invariably adds to the concentration of ARGs present in these foods [118]. When humans consume foods from these sources, they risk the transfer of ARG carrying bacteria into their gut microbiome, thus adding to the pool of resistant genes and elevating ARG infection risks [119]. Although previous studies have examined and promoted the role of animal protein sources in infant nutrition (as complementary foods) to fill the nutrient deficiencies observed in breast milk when infants’ nutritional needs increase during weaning [120,121], research has associated consumption of red meat in adults to increased carriage of multidrug-resistant *E. coli* [122]. Therefore, weaning diet has the potential to have both positive and negative effects on infant carriage of ARGs, and more research is needed to understand the extent to which fiber and animal product consumption interact to shape the resistome.

## 7. Infant Gut Resistome as a Source of ARGs

Not all ARGs are of equal concern for public health; genes on mobile genetic elements are of greater concern than chromosomal resistance genes [42,104,123,124]. ARGs on mobile genetic elements can undergo HGT from one microorganism to another [23]. ARGs on mobile genetic elements in biomes with greater bacterial diversity and density are more likely to undergo HGT [125]. As the infant gut microbiome is enriched for ARGs and is more likely to come in contact with human-relevant pathogens than other biomes (e.g., the soil biome), the infant gut microbiome is a potential hotspot for ARG transmission. The infant gut microbiome can potentially serve as a source of nosocomial ARGs, for example, in inhalable dust [126]. There is also an increased chance for transmission of resistant pathogens in childcare facilities as young children are immunologically naïve and at greater risk of infection generally, and there are multiple outbreak reports of antimicrobial-resistant organisms occurring in daycares [127].

## 8. Conclusions

The intricate interaction between ARGs, the gut microbiome, and infant nutrition calls for more investigation to develop strategies that are efficacious and capable of rapidly arresting AMR spread. As expounded here, the neonatal period through the introduction of complementary foods represents a critical window of time during which the infant gut microbiome develops. This makes it a critical time period for the establishment of a healthy gut microbial community. Because the gut resistome is part of the gut microbiome, the establishment of the gut microbiome can have a significant influence on ARG carriage. Of particular importance is the possibility that diet can help to shape the resistome and thereby control the spread of ARGs. Early evidence indicates that dietary differences are associated with differences in the gut resistome and carriage of ARGs, but further research is needed to tease apart the complex relationship between diet and ARG carriage before specific recommendations for a diet to reduce ARG carriage can be made. This is especially true since, as previously discussed in this paper, dietary fiber is associated with decreased ARG levels and meat consumption with increased ARG infection risk, but there is not a clear difference in ARG carriage between omnivores and vegans.

To enable future recommendations, more original studies seeking to elucidate the underlying mechanisms and role of diet in infant acquisition and transmission of ARGs and to understand the health implications of a changing resistome over time are needed. Such studies should consider detailed analysis of different dietary components. For example, are fibers from specific foods associated with decreased ARG carriage? Consumption of which animal products are associated with the introduction of new ARGs? Future studies should also emphasize the need for multidisciplinary approaches to holistically establish the links between ARGs, the infant gut microbiome, and nutrition through the lens of the One Health approach.

## Data Availability

This is a review article. No new data was generated for this manuscript.
